# The Development and Preliminary Validation of a Rhythmic Jumping Task for Coordination Assessment: A Task Design Based on Upper and Lower Limb Motor Congruency

**DOI:** 10.3390/jfmk10030261

**Published:** 2025-07-11

**Authors:** Runjie Li, Tetsuya Miyazaki, Tomoyuki Matsui, Megumi Gonno, Teruo Nomura, Toru Morihara, Hitoshi Koda, Noriyuki Kida

**Affiliations:** 1 Doctoral Programs of Biotechnology, Graduate School of Science and Technology, Kyoto Institute of Technology, Hashikami-cho, Matsugasaki, Sakyo-ku, Kyoto 606-8585, Japan; draculalee@icloud.com (R.L.); mtsports0512@gmail.com (T.M.); 2 Marutamachi Rehabilitation Clinic, 12 Nishinokyo Kurumazakacho, Nakagyo-ku, Kyoto 604-8405, Japan; matsui.tomoyuki.sports.reha@gmail.com (T.M.); toru4271@koto.kpu-m.ac.jp (T.M.); 3 Faculty of Arts and Sciences, Kyoto Institute of Technology, Hashikami-cho, Matsugasaki, Sakyo-ku, Kyoto 606-8585, Japan; h-koda@kit.ac.jp; 4 Department of Childhood Education, Faculty of Childhood Education, Nagoya Aoi University, 3-40 Shioji-cho, Mizuho-ku, Nagoya-shi 467-8610, Japan; gonno@nagoya-aoi.ac.jp; 5 Department of Health and Sports Sciences, Faculty of Health and Medical Sciences, Kyoto University of Advanced Science, 1-1 Nanjootani, Kameoka, Kyoto 621-8555, Japan; note0420@gmail.com

**Keywords:** motor coordination, rhythm jump, task structure, inter-limb interaction, movement analysis, evaluation feasibility

## Abstract

**Background:** The coordination between the upper and lower limbs is essential for athletic performance. However, the structural features that influence coordination difficulty remain insufficiently understood. Few studies have systematically analyzed how task components such as the directional congruence or rhythm structure affect inter-limb coordination. **Objective:** This study aimed to clarify the structural factors that influence the difficulty of upper–lower limb coordination tasks under rhythmic constraints and to explore the feasibility of applying such tasks in future coordination assessments. **Methods:** Eighty-six male high school baseball players performed six Rhythm Jump tasks combining fixed upper limb movements with varying lower limb patterns. The task performance was analyzed using three indices: full task success, partial success, and average successful series. One year later, a follow-up test involving 27 participants was conducted to evaluate the reproducibility and sensitivity to the performance change. **Results:** The task difficulty was significantly affected by structural features, including directional incongruence, upper limb static holding, and rhythmic asynchrony. The tasks that exhibited these features had lower success rates. Some tasks demonstrated moderate reproducibility and captured subtle longitudinal changes in the performance. **Conclusions:** The results highlight the key structural factors contributing to coordination difficulty and support the potential applicability of Rhythm Jump tasks as a basis for future assessment tools. Although further validation is necessary, this study provides foundational evidence for the development of practical methods for evaluating inter-limb coordination.

## 1. Introduction

### 1.1. Motor Coordination

Motor coordination has been identified as a fundamental component of human motor skills [1,2,3]. This term refers to the process of coordinating the movements of various body parts to achieve a specific intended motion. In the domain of biomechanics, motor coordination is defined as the synchronized movement of multiple body parts to accomplish goal-directed actions such as walking [4]. The significance of motor coordination in rhythmic gymnastics has been elucidated in the field of motor learning research [5]. Similarly, the development of adequate motor coordination is reported to contribute to the optimization of athletic skills [3]. These studies provide valuable insights into the significance of motor coordination in enhancing sports performance.

A longitudinal study involving children in the Azores Islands suggested that motor coordination is an important predictor of physical activity during childhood [6]. Additionally, it has been argued that motor coordination should not be regarded solely as a general human function, but rather as a complex and sophisticated system that plays an essential role [7]. In summary, coordination is a prerequisite for athletes to use their bodies in complex and refined ways.

The research on motor coordination is extensive, including studies on bimanual coordination [8,9] and eye–hand coordination [10,11,12,13]. Research has also been conducted on upper–lower limb coordination [14,15]. Such coordination has been observed not only in fundamental motor skills, such as walking and jumping, but also in sports such as volleyball, rhythmic gymnastics, dance, and martial arts. Although not universally essential for participation in all sporting activities, motor coordination is regarded as a favorable characteristic for enhancing athletic performance. For instance, motor coordination has been identified as a key predictor of elite-level success among female volleyball players, in addition to height and jumping ability [16]. This suggests that, while a high coordination is not always essential, enhancing it can substantially contribute to improved performance. Consequently, the research on motor coordination is of considerable relevance for understanding athletic skill acquisition.

### 1.2. Motor Coordination Training

In recent years, there has been an increasing focus on the efficacy of motor coordination training as a means of enhancing motor skills and athletic performance. It is hypothesized that enhancements in coordination will not only facilitate skill acquisition and athletic performance but also contribute to injury prevention [17,18]. This tendency is particularly pronounced in studies focusing on athletes and children. For instance, it was reported that the introduction of jump rope training in preadolescent soccer players resulted in improved overall motor skills and coordination [19]. A similar study reported that regular motor coordination training in male adolescents aged 15–17 years enhanced athletic performance [20]. The findings of this study indicate that effective motor coordination training can contribute to athletic development.

However, to accurately assess the effects of such training, it is essential to establish appropriate evaluation methods. In the context of strength training among high school athletes, the use of standardized assessment tools is critical for the proper interpretation of training effects [21,22]. In the absence of such tools, the reliance on the subjective judgment or experience of instructors may result in an inaccurate evaluation of outcomes.

Consequently, the regular evaluation of athletes’ coordination abilities during training may facilitate the optimization of their training regimens and future objectives. The present study was grounded in the extant literature, and its objective was to evaluate motor coordination. The primary objective of this study was to assess upper and lower limb coordination. However, given the paucity of existing methods for evaluating such coordination, our initial objective was to explore evaluation methods that specifically target these areas.

### 1.3. Evaluation Methods

In addition to the development of motor coordination training, there has been a recent increase in the focus on evaluation methods that can accurately assess coordination ability. For instance, motor coordination in child gymnasts was measured using a force plate [23]. Methods requiring sophisticated equipment are frequently impractical in everyday settings, such as training grounds or educational contexts. Consequently, the demand for simple and accessible evaluation tools has increased.

In response to this challenge, coordination assessment methods for children that utilize simple tasks such as hopscotch, single-leg standing, or mini-hurdle runs are proposed [24,25,26]. The utilization of these methodologies confers several advantages, including enhanced accessibility and optimized time efficiency, obviating the need for specialized equipment. Nevertheless, these devices are primarily designed for children in their developmental stages, and their applicability and precision in healthy adults or athletes remain limited [27].

Moreover, the majority of contemporary simple assessments concentrate on specific subsystems, such as hand–eye coordination or the independent functioning of either the upper or lower limbs. However, few tools comprehensively evaluate the simultaneous coordination between the upper and lower limbs. This underscores the necessity for a novel assessment method that can systematically and efficiently capture upper–lower limb coordination.

The present study is informed by this recognition, and its focus is on “Rhythm Jump”, a concept proposed by the Rhythm Training Association [28]. Rhythm Jump is defined as a movement pattern characterized by independent yet simultaneous movements of the upper and lower limbs in accordance with a fixed tempo. The substance under discussion is characterized by its ability to facilitate natural coordination. Furthermore, the task difficulty can be adjusted by modifying the combinations of movement patterns, allowing flexible applications tailored to individual ability levels.

In view of the aforementioned background, the subsequent section delineates the specific objectives of the present study.

### 1.4. The Purpose of the Study

Based on the Rhythm Jump framework [28], this study aimed to develop and evaluate a set of rhythm-based tasks designed to assess upper–lower limb motor coordination. The tasks were systematically structured by fixing upper limb movements and varying lower limb jumping patterns, while integrating elements such as static upper limb control and directional incongruency to elicit coordination demands.

The aims of the present study are threefold.

First, to analyze performance outcomes—including success rates and error tendencies—across the six tasks and clarify how specific structural components, such as static upper limb control, directional congruency, and rhythm features, contribute to coordination success or failure.

Second, to examine the consistency between the theoretically assumed task difficulty levels and actual participant performance, thereby evaluating and refining the criteria for future task classification.

Third, to explore the feasibility of using the developed task set as an assessment tool for upper–lower limb motor coordination through a follow-up measurement conducted one year after the initial test, focusing on the reproducibility and sensitivity to developmental or training-related changes.

Through this structural manipulation and longitudinal design, this study seeks to provide foundational knowledge for developing a reliable, scalable, and practically applicable coordination assessment tool.

## 2. Materials and Methods

### 2.1. Participants

A total of 86 male athletes (height: 169.3 ± 6.1 cm; body mass: 66.3 ± 7.1 kg; mean age: 16.2 ± 0.7 years) belonging to high school baseball teams participated in this study. All participants engaged in regular sports-specific training activities, including base running, batting, and pitching. However, they did not participate in any training specifically targeting upper and lower limb coordination, which was evaluated in this study. None of the participants reported a history of musculoskeletal or neurological disorders.

Among them, 31 participants consented to participate in a follow-up evaluation approximately one year after the initial test. Of these, 27 (height: 170.2 ± 5.2 cm; body mass: 67.0 ± 7.0 kg; mean age 16.7 ± 0.4 years) completed the follow-up, while 4 withdrew from the study for personal reasons. Despite the absence of structured coordination training during this period, it is hypothesized that natural growth, development, and habitual physical activity may have exerted a certain influence on athletes’ upper and lower limb coordination abilities.

As this was an exploratory study, no a priori sample size calculation was conducted. Instead, the sample size was determined based on the number of eligible participants available during the data collection period. To reduce variability in motor experience and ensure a relatively homogeneous training background, all participants were selected from a single high school boys’ baseball team. This sampling strategy was chosen to enhance the internal validity of the findings by minimizing differences in sport-specific coordination experience.

### 2.2. Task and Movement Description

In this study, the task involved repeated light jumps synchronized with a metronome beat. The momentary static posture adopted at each metronome beat was designated as “posture”, while the movements of the upper and lower limbs occurring between beats were defined as “motion”. The series under consideration comprised four motions, with each individual task consisting of four consecutive series.

The task structure involved laterally symmetrical movement patterns constrained to the frontal plane to enable controlled observation of upper and lower limb coordination specific to this plane.

**Figure 1:**
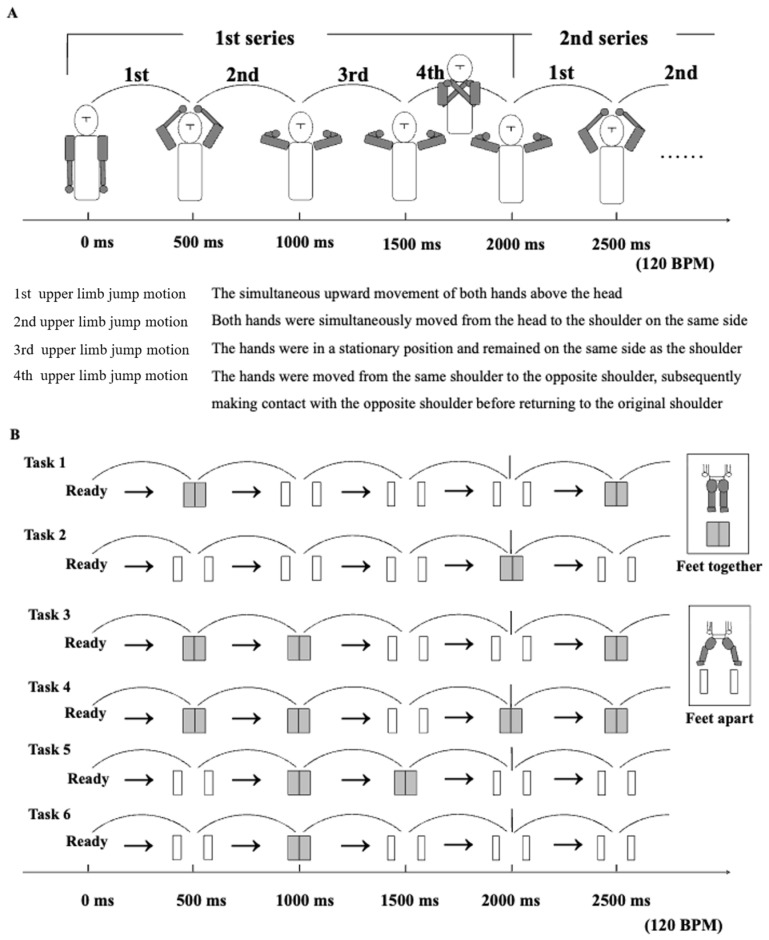
Images of the jump motion of the upper and lower limbs and six types of tasks. (**A**) The jump motion of the upper limbs. (**B**) The jump motion of the lower limbs.

The upper limb motion was consistent across all tasks, as shown in Figure 1A. In the first motion, both hands moved in unison, moving upward symmetrically and converging toward the center of the head in the frontal plane, and were situated directly above the head. Typically, the hands were moved from a position slightly above the ipsilateral shoulder joints to the top of the head. However, only during the first motion of the first series did the movement start from a preparatory position, with the arms hanging down at the sides of the body. In the second motion, both hands descended simultaneously from the center above the head to a position above the ipsilateral shoulder joint. During the third motion, both hands remained above the ipsilateral shoulder. In the fourth motion, both hands moved rapidly from the ipsilateral shoulder to the contralateral shoulder, briefly crossed over, touched, and then promptly returned to the ipsilateral shoulder. The same sequence was repeated in the second cycle, with both hands moving upward symmetrically and converging toward the center of the head in the frontal plane to initiate the next cycle.

With regard to the lower limbs, as illustrated in Figure 1B, two fundamental postures were delineated: “feet-apart” and “feet-together”, referring to the positioning of the feet relative to each other. Four fundamental lower limb motions were defined based on these two postures. Transitioning from a feet-together position to a feet-apart position was referred to as a “feet-apart motion”, while transitioning from a feet-apart position to a feet-together position was referred to as a “feet-together motion”. Maintaining the feet-apart posture was termed a “keeping feet apart motion”, and maintaining the feet-together posture was termed a “keeping feet together motion”. These motions were then combined to create six lower limb patterns, each corresponding to upper limb movements in the respective tasks.

The six tasks were selected based on preliminary experiments. In the preliminary testing, 16 types of lower limb patterns were evaluated. It was found that performing all the patterns placed an excessive burden on the participants. Additionally, four patterns that were successfully performed by all participants after a brief practice session were considered unsuitable for evaluation because of their low level of difficulty and were used only as introductory tasks. The remaining twelve patterns were grouped into six pairs and one pattern from each pair was selected, resulting in a final set of six tasks.

During task performance, the metronome tempo was set at 120 beats per minute (bpm) to allow the participants to move naturally and comfortably. This tempo was within the range (100–120 bpm) typically found to be comfortable for adults [29] and corresponded to an interval of approximately 500 milliseconds per beat. In the task, jumps, landings, postures, and movement transitions occurred continuously at 500-millisecond intervals, so that a series was completed in approximately 2000 milliseconds (2 s). Each task consisted of four consecutive repetitions of the same pattern series with no rest periods between movements, thus requiring continuous rhythmic performance. Details of the upper and lower limb postural changes and motion transitions are shown in Figure 1A and Figure 1B, respectively.

### 2.3. Implementation Procedure

First, the tasks involved in conducting the study were explained. We explained the positions and movements of the upper and lower limbs, as well as the meaning of “series”, using verbal explanations and demonstrations. Participants then practiced the upper and lower limb motions individually to confirm their understanding of each movement.

The participants performed an introductory task in which they jumped with their feet together and then with their feet apart to beat a metronome set at 120 bpm. The purpose of this task was to confirm whether the participants understood the coordinated motions of their upper and lower limbs.

The participants were then given five minutes of free practice time across the six tasks used in the experiment. During free practice, the subjects were allowed to repeat any task they wanted, regardless of whether they succeeded or failed. We also allowed “imagery practice”, in which the participants imagined the movements in their minds without actually performing them. In terms of practice time, it is thought that a balance between practice and rest is an important factor in motor learning, and it has been suggested that excessive practice may reduce learning efficiency. In particular, it was reported that the combination of practice and rest influenced the acquisition of tracking skills and that short periods of focused practice were effective [30]. In this study, we set the free practice period to 5 min, considering both these findings and the time constraints of the experiment as a whole.

After the free practice, the six main tasks were performed. Each task was presented in a predetermined order, and the next task was presented after completion. Short breaks were allowed between tasks; however, no practice or interruptions were allowed during the tasks. Each task was performed only once, and the measurements were taken when all six tasks were completed.

### 2.4. Data Processing

The data used in this study were composed entirely of video recordings taken during the testing sessions for all participants. The accuracy of each participant’s motion was assessed through visual observation and motion analysis. If the motion trajectories of the upper limbs, the sequence of lower limb motions, or the postures at landing deviated from the prescribed motion patterns, the motion was judged as a “failure”, even if only a single motion or posture failed to meet the criteria. If any failure occurred within a series of motions, the entire series was considered a failure. All video data were independently verified by each researcher, and the recorded content was cross-checked to ensure data accuracy.

The video recordings were captured using a digital video camera (GC-YJ40, JVC Kenwood Corporation, Yokohama, Japan; manufactured in Malaysia). During the experimental sessions, motion playback was conducted using a personal computer (MacBook Pro, Apple Inc., Cupertino, CA, USA) with the preinstalled default media player on macOS (QuickTime Player). No additional software was used.

To facilitate analysis, data processing was divided into two stages: “motion evaluation” and “evaluation index assessment”. In the motion evaluation stage, attention was focused on the first series, which was experienced first by all the participants, and success rates were analyzed in detail based on the execution status of the upper limb motions. The target consisted of five motions: four in the first series, starting from the preparatory posture at the beginning of the task, and the first motion in the second series. For each motion, we determined whether the upper limb performed the prescribed trajectory and timing, and the number and percentage of successful motions were calculated.

In addition to the success rates for individual motions, cumulative success rates were calculated using a forward accumulation method, such as “continuous success for the first and second motions”, “continuous success for the first through third motions”, “continuous success for all four motions in the first series”, and “continuous success for all five motions, including the initial motion of the second series”. Statistical tests for population proportions at a 5% significance level were used for comparisons. It should be noted that although all six tasks used in this study shared the same basic upper limb motions, each task was composed of a combination of different lower limb motions, as shown in Figure 1. Figure 1 lists the lower limb motion patterns combined with the five upper limb motions for each task, allowing visual confirmation of task-specific characteristics and differences in difficulty.

Three different evaluation indices were established to assess the task performance abilities of all participants. First, the successful completion of all four series (complete performance) was used as the main index to record the proportion of participants who successfully completed all series within a single task. This index assesses the ability to complete a task perfectly and can be used in test scoring and immediate feedback contexts. Second, the achievement of “success in at least one series” was used as a subsidiary index, recording the proportion of participants who succeeded in at least one of the four series within a task. This made it possible to distinguish between participants who were able to partially perform the task and those who were unable to perform it. Cochran’s Q test was used to compare success rates between tasks at a 5% level of significance. However, only *p*-values are reported in the main text of the Results section, and the Q statistics are omitted. Third, the “average number of successful series within a task” was used as a supporting index by calculating the mean number of successful series for each task, allowing a quantitative evaluation of task performance levels. Comparisons of the mean number of successful series between the different tasks were performed using paired *t*-tests at a 5% level of significance.

In addition, the same 27 participants were measured twice, approximately one year apart, to examine changes in their ability to perform the tasks. The three indices mentioned above (“success in all four series”, “success in at least one series”, and “average number of successful series”) were used to compare the first and second measurements for the same task. For the two binary variables (“success in all four series” and “success in at least one series”), differences between the first and second measurements for the same task were analyzed using McNemar’s test at a 5% significance level. Cramér’s V was calculated to assess the degree of association with task performance. For the “average number of successful series”, a paired *t*-test was performed at a 5% significance level to determine whether there were significant differences between the first and second measurements for the same task. In addition, Spearman’s rank correlation coefficient (Spearman’s ρ) was calculated to quantify the relationship between the first and second results for each index. These analyses were used to comprehensively examine changes in task performance along with the reproducibility and reliability of the results.

## 3. Results

### 3.1. Success Rates for Each Combination of Upper and Lower Limb Motions

**Figure 2:**
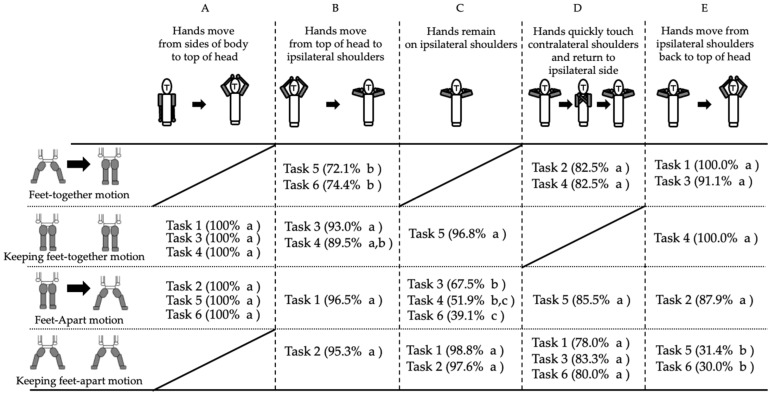
This table presents a comparison of the motions across the six tasks, where the comparisons were conducted vertically within columns rather than horizontally across rows. Letters such as “a” or “b” are used to indicate the results of population proportion tests for differences in proportions. A value labeled “a” was significantly higher than a value labeled “b” (*p* < 0.05). No significant differences were observed between values with the same letter. The notation “a, b” or “b, c” indicates that no significant difference was found between those values.

In this analysis, upper limb motions were held constant across all tasks, and success rates were compared based on different combinations of lower limb motions associated with each task. Figure 2 shows the success rates for each combination of upper and lower limb motions across the six tasks, as well as the results of the comparisons across tasks.

Figure 2A shows a comparison of the success rates for the motion of moving both hands from the preparatory posture to the center above the head. For all tasks, the success rate was 100% and there were no significant differences between the tasks.

Figure 2B compares the success rates for the motion of lowering both hands from above the head to the ipsilateral shoulder. Tasks 1 to 3 had significantly higher success rates than Tasks 5 and 6 (χ^2^ = 43.836, *p* < 0.001). No significant differences were observed among Tasks 1 to 3, between Tasks 5 and 6, or among Task 4 and the other tasks.

Figure 2C shows a comparison of the success rates for maintaining both hands on the ipsilateral shoulder. Tasks 1, 2, and 5 showed significantly higher success rates than Tasks 3, 4, and 6 (χ^2^ = 135.121, *p* < 0.001). No significant differences were observed between Tasks 1, 2, and 5 or between Tasks 4, 3, and 6. However, Task 6 exhibited a significantly lower success rate than Task 3.

Figure 2D shows a comparison of the success rates for the motion of quickly crossing both hands from the ipsilateral shoulder to the contralateral shoulder and from the back to the original shoulder. No significant differences were found between the tasks (χ^2^ = 1.368, *p* = 0.928).

Finally, Figure 2E shows a comparison of the success rates for the motion of moving both hands from the ipsilateral shoulder back to the center above the head. Tasks 1 to 4 showed significantly higher success rates compared to Tasks 5 and 6 (χ^2^ = 127.655, *p* < 0.001). However, no significant differences were observed among Tasks 1 to 4 or between Tasks 5 and 6.

### 3.2. The Comparison of the Task Performance Across the Six Tasks

**Figure 3:**
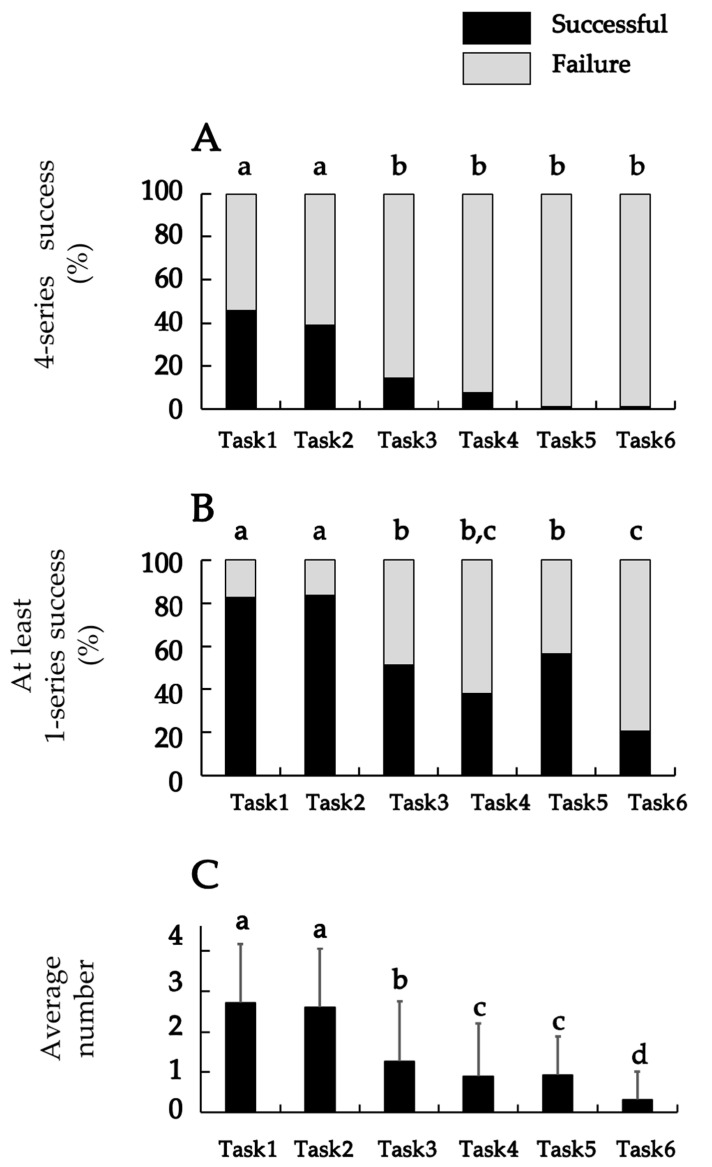
The comparison between the six tasks. (**A**) The percentage of success with four series; (**B**) the percentage of success with at least one series; and (**C**) the average number of successful series. Results of (**A**,**B**) using Cochran’s Q test and the results of (**C**) using the *t*-test, those are presented in the form of “a”, “b”, ”c”, and “d”. The value indicated by “a” is significantly higher than “b”, “c”, and “d”. Similarly, the value indicated by “b” is significantly higher than “c” and “d”. The value indicated by “c” is significantly higher than “d”. The value indicated by “b, c” indicates that no significant difference was detected between “b” and “c” (*p* < 0.05).

Comparisons across the six tasks are shown in Figure 3. Figure 3A shows the proportion of participants who achieved success based on the criterion of completing all four series together with the results of the comparisons between tasks. The success rates for Tasks 1 (45.7%) and 2 (39.1%) were significantly higher than those for Tasks 3 (11.1%), 4 (3.7%), 5 (0.0%), and 6 (0.0%) (all *p* < 0.001). There was no significant difference between Tasks 1 and 2.

Figure 3B shows the proportion of participants who achieved success based on the criterion of completing at least one series together with the results of the comparisons between tasks. The success rates for Tasks 1 (82.6%) and 2 (83.7%) were significantly higher than those for the other tasks (all *p* < 0.001). However, the success rate for Task 6 was the lowest of all tasks and was significantly lower than those for Tasks 1 (*p* < 0.001), 2 (*p* < 0.001), 3 (*p* < 0.001), and 5 (*p* < 0.001). No significant differences were found between Tasks 6 and 4 (*p* = 0.145) or between Tasks 3, 4, and 5.

Figure 3C shows the mean number of successful series for each task and the results of the comparisons between the tasks. The mean number of successful series for Task 1 (2.72 ± 1.48 series) and Task 2 (2.62 ± 1.44 series) was significantly higher than for the other tasks (all *p* < 0.001), with no statistically significant difference between Task 1 and Task 2 (*p* = 0.541). Meanwhile, the mean number of successful series for Task 6 was the lowest of all tasks and was significantly lower than those for Tasks 1 (*p* < 0.001), 2 (*p* < 0.001), 3 (*p* < 0.001), 4 (*p* < 0.001), and 5 (*p* < 0.001). In addition, the mean number of successful series for Tasks 4 and 5 was significantly lower than that of Task 3 (Task 4: *p* = 0.017; Task 5: *p* = 0.024), although no significant difference was observed between Tasks 4 and 5 (*p* = 0.856).

### 3.3. Comparison of First and Second Session Participants

**Figure 4:**
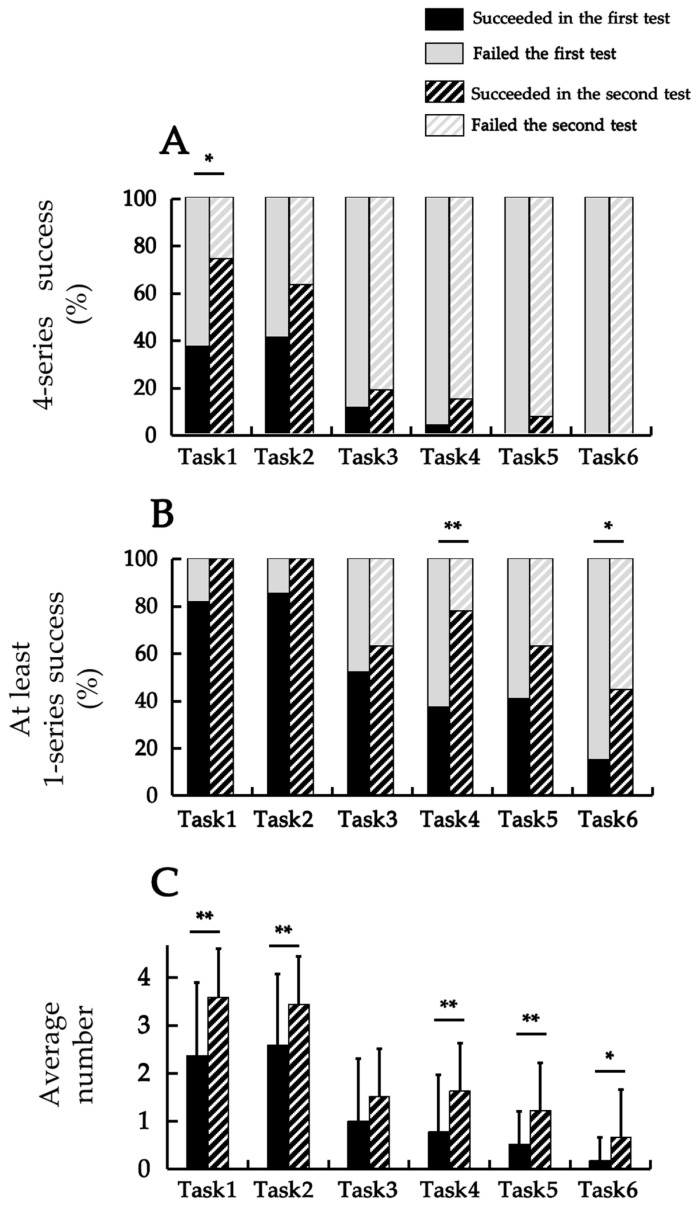
The comparison of the performance between the first and second tests for the same participants. (**A**) The change in the success rate for four series, analyzed using the McNemar test; (**B**) the change in the success rate for at least one series, analyzed using the McNemar test; (**C**) the change in the average number of successful series, analyzed using the paired *t*-test (* *p* < 0.05; ** *p* < 0.01).

Figure 4 summarizes the comparison of performance between the first and second sessions for the same participants across the six tasks. Figure 4A illustrates the success rates based on the criterion of success in all four series, which were compared using McNemar’s tests. For Task 1, a significant increase was observed from the first measurement (37.0%) to the second (74.1%) (χ^2^ = 5.353, *p* = 0.002). In contrast, no significant differences were found for Tasks 2 (χ^2^ = 2.725, *p* = 0.109), 3 (χ^2^ = 4.993, *p* = 0.625), or 4 (χ^2^ = 5.750, *p* = 0.250). For Tasks 5 and 6, no statistical tests could be conducted because of the very small or nonexistent number of successful participants in both the first and second measures.

Figure 4B presents the success rates based on the criterion of success in at least one series, analyzed using McNemar’s test. No significant changes were found for Tasks 3 (χ^2^ = 2.925, *p* = 0.508) or 5 (χ^2^ = 0.003, *p* = 0.180), whereas significant increases were observed for Tasks 4 (χ^2^ = 1.322, *p* = 0.003) and 6 (χ^2^ = 0.057, *p* = 0.039). For Tasks 1 and 2, all the participants were successful in the second measurement (100%); therefore, statistical testing could not be performed.

Figure 4C displays the average numbers of successful series, which were compared using paired *t*-tests. Significant increases were observed for Task 1 (*t* = −4.845, *p* = 0.001), Task 2 (*t* = −2.979, *p* = 0.006), Task 4 (*t* = −3.507, *p* = 0.002), Task 5 (*t* = −3.124, *p* = 0.004), and Task 6 (*t* = −2.565, *p* = 0.016). Although there was a trend towards improvement in Task 3, no significant difference was observed (*t* = −1.763, *p* = 0.090).

The associations between the first and second measures were examined. First, based on the results of Cramér’s V for success in all four series, significant associations were found for Tasks 1 (V = 0.454, *p* = 0.018), 3 (V = 0.438, *p* = 0.023), and 4 (V = 0.470, *p* = 0.015). No significant association was found for Task 2 (V = 0.324, *p* = 0.093). No statistical tests were performed for Tasks 5 and 6.

Next, based on the results of Cramér’s V for success in at least one series, no significant associations were found for Tasks 3 (V = 0.335, *p* = 0.081), 4 (V = 0.225, *p* = 0.241), 5 (V = 0.012, *p* = 0.952), and 6 (V = 0.047, *p* = 0.809). Statistical tests could not be performed for Tasks 1 and 2 because all the participants succeeded in the second measurement.

Finally, the results of Spearman’s rank correlation coefficients for the average number of successful series are presented. Significant positive correlations were found for Tasks 1 (*ρ* = 0.550, *p* = 0.003), 2 (*ρ* = 0.382, *p* = 0.049), 3 (*ρ* = 0.443, *p* = 0.021), and 4 (*ρ* = 0.464, *p* = 0.015). In contrast, no significant correlations were found for Tasks 5 (*ρ* = 0.257, *p* = 0.196) or 6 (*ρ* = 0.030, *p* = 0.883).

## 4. Discussion

### 4.1. Exploring Coordination Challenges in Different Upper–Lower Limb Combinations

The task involving the movement of both hands from the preparatory position to the center above the head was performed successfully by nearly all participants across all conditions. This can be attributed to the fact that the motion was executed in a stable preparatory posture, which allowed sufficient time for anticipatory planning and motor preparation. Previous research has highlighted the role of preparatory control in motor learning, indicating that, even in the absence of physical execution, repeated cognitive rehearsal can contribute to improved motor performance [31]. In the present study, the preparatory stance involved standing still with the feet together, and the subsequent lower limb movements were limited to either a feet-apart jump or a feet-together jump, making the prediction of movement sequences relatively straightforward. This action occurred only once, at the onset of the first series.

Next, attention was directed to the combinations in which the upper limbs moved laterally from the center above the head toward the ipsilateral shoulder. Task 1, which combined this upper limb motion with a feet-apart lower limb pattern, had the highest success rate. This alignment features outward movements of both limb sets, resulting in a high degree of directional congruence. This congruence reduces neural interference and supports stable motor execution in coordinated tasks [32]. In contrast, Tasks 5 and 6, which involved a lower limb movement toward a feet-together position during the same upper limb motion, showed substantially reduced success rates. The mismatch in the movement direction between the limbs in these tasks likely disrupts coordination by increasing the cognitive and neural demands associated with managing directional incongruence, a concept that has been described in relation to directional constraints [32,33].

The tasks involving static upper limb postures, where the participants maintained their hands at the ipsilateral shoulders, also revealed performance differences based on the accompanying lower limb motions. Tasks 3, 4, and 6, which required dynamic transitions in the lower limb stance while maintaining upper limb stillness, showed relatively low success rates. Conversely, Tasks 1, 2, and 5, in which a consistent foot position was maintained during the same upper limb posture, exhibited a comparatively higher performance. These findings suggest that changes in the lower limb configuration can interfere with upper limb stability, likely through inter-limb neural linkages. Previous studies have demonstrated that rhythmic movements in one limb may involuntarily elicit activity in the other limbs, even when those limbs are intended to remain stationary [34,35]. This implies that the interference effects extend beyond spatial dynamics and may involve rhythm- or entrainment-related influences.

A further increase in the task complexity was observed in combinations involving upper limb crossing motions, in which the hands moved sequentially from the ipsilateral shoulder to the contralateral shoulder and returned. This motion involves two distinct directional changes within a single jump cycle, introducing substantial spatial and temporal complexities. Across all tasks, the success rate for this motion was consistently low, and the task-to-task variation was minimal. This pattern indicates that the difficulty stemmed not from differences in the lower limb structure but from the inherent complexity of the upper limb movement itself. It is well established that coordinated limb movements tend to stabilize under one-to-one rhythmic conditions between limbs [33,36]. In contrast, the crossing motion imposes a two-to-one rhythm ratio by introducing asynchronous temporal dynamics. This rhythmic incongruence challenges the timing regulation of the central nervous system, thereby reducing automatic coordination. Motor control models suggest that asynchronous rhythms require the brain to simultaneously manage multiple timing mechanisms, which increases the cognitive load and diminishes performance [37,38,39].

Finally, attention was turned to the movement of the upper limbs, returning from the ipsilateral shoulders to the center above the head. This motion, which represents the mirror image of the earlier lateral movement, requires the arms to move inward while the lower limb patterns vary. The results indicated that Tasks 1 through 4 had substantially higher success rates than Tasks 5 and 6. In the latter two tasks, the participants were required to maintain a feet-apart lower limb posture while simultaneously performing an inward arm motion. This mismatch in the directional intent between the limb sets likely increases neural interference, resulting in diminished coordination stability [32,33]. Although Task 2 also included a feet-apart stance, its success rate was slightly lower than those of Tasks 1, 3, and 4, all of which involved a feet-together configuration, suggesting that even minor directional conflicts can impair motor coordination.

In summary, the findings from this section indicate that three primary structural elements—directional congruence, rhythmic synchrony, and static control—are crucial determinants of success in upper–lower limb coordination tasks. Coordinated movement was facilitated when both limbs moved in the same direction, either inward or outward, because this alignment minimized neural interference. In contrast, directional mismatches introduce substantial cognitive demands that hinder automatic coordination. Similarly, a synchronous one-to-one rhythm between limb movements enhances stability, whereas asynchronous patterns, particularly those with a two-to-one structure, significantly decrease success rates [36,37,38]. These results underscore the importance of task design in motor coordination assessments and highlight the potential value of deliberately manipulating structural features to match the learners’ abilities and training objectives.

### 4.2. Evaluating Structural Contributions to Task Difficulty Across the Six Tasks

Clear differences in the success rates were observed across the six tasks. Tasks 1 and 2 yielded comparatively higher success rates, whereas Task 6 consistently yielded the lowest performance across all evaluated indicators. Nevertheless, even in Task 1, the most successful condition, fewer than half the participants managed to complete all four series, indicating that sustaining stable coordination between the upper and lower limbs remains inherently demanding. One structural factor likely contributing to this difficulty is the upper limb crossing motion, which is a two-phase movement included in all tasks. Given its spatial and temporal complexity, this component plausibly imposed a baseline level of difficulty and functioned as a fundamental constraint on the overall performance, as suggested by prior research on coordination control under spatial constraints [33,40]. Based on this premise, the following analysis focuses on additional structural factors beyond this shared element that may have further influenced the task difficulty.

In Task 1, apart from the crossing motion, the upper and lower limbs moved in congruent directions, and the stance width remained largely constant in the open-leg position. These characteristics likely contributed to smoother coordination control. Task 2 involved minimal changes in the stance width and a predominantly congruent movement direction. However, in the final jump of Task 2, the upper limbs moved inward, whereas the lower limbs transitioned from an open to a closed position, thereby introducing a brief phase of directional incongruence. Even this momentary disruption may have interfered with automatic motor control, reducing the Four-Series Success Rate in comparison to Task 1, as previously demonstrated in studies examining the effects of movement congruency on motor fluency [41,42].

Tasks 3 and 4 demonstrated moderately higher success rates than Tasks 5 and 6. Both tasks shared a key structural feature in which, during the static “keep” posture of the upper limbs, the lower limbs shifted from a closed to an open position, resulting in a temporary directional misalignment. Although several participants managed to complete at least one series in Task 5, sustaining a stable coordination across all four series proved difficult, leading to a low overall success rate. This challenge can be attributed to the alternation between open and closed stances, which requires the constant reorganization of motor sequences and the real-time control of movement timing. Frequent reversals in the movement direction also appear to increase the demands on selective attention and motor planning, thereby accumulating a cognitive load and compromising coordination [43,44].

Task 6 exhibited the lowest overall success rate. The directional congruence between the upper and lower limbs was largely absent, and the upper limb stillness phases were frequently disrupted by dynamic transitions in the lower limbs. These overlapping structural demands likely reduced the automaticity of motor control and impaired the performance stability. Prior findings have shown that tasks involving multiple conflicting movement cues can deplete internal attentional resources, which in turn diminish performances in complex motor tasks [40,45]. The cumulative nature of such structural loads offers an insight into the fundamental challenges in executing high-level coordination under compounded interference.

Moreover, the structural configurations employed in this study suggest that the coordination difficulty can be systematically modulated through an intentional task design. Specifically, adjusting factors such as the movement direction alignment and the frequency of directional transitions allows for fine-grained control over the task complexity. Adaptability enhances the educational and evaluative potential of task systems. Furthermore, design flexibility aligns well with motor learning frameworks that emphasize task difficulty matching as a core principle for optimizing skill acquisition [46]. For instance, simpler configurations, such as those in Tasks 1 and 2, may provide early learners with opportunities for early success and confidence building, whereas more complex structures, such as Tasks 5 and 6, may challenge advanced learners to refine and consolidate adaptive motor strategies.

This section examines the influence of structural differences across tasks on the coordination stability and perceived difficulties. While the crossing motion served as a foundational source of spatial complexity, additional elements, including the directional congruence between limbs, the timing and frequency of stance changes, and the presence of motion reversals, exerted compounding effects on performance. Notably, even with identical upper limb movements, variations in lower limb configurations modulated the degree of neural interference and ultimately affected the coordination stability. The ability to systematically manipulate these structural parameters demonstrates the potential of this task framework for individualized and developmentally appropriate designs in both instructional and evaluative contexts.

### 4.3. The Reproducibility of the Task Performance in the Same Participants

The results of this study indicated that, among the various evaluation metrics employed, a significant improvement in the “Four-Series Success Rate” was observed only for Task 1 at the one-year follow-up. No significant differences were detected for Tasks 2, 3, or 4, whereas statistical analyses for Tasks 5 and 6 were not feasible because of the extremely low number of participants who achieved success in both the initial and follow-up assessments. Notably, none of the participants underwent specialized upper–lower limb coordination training during the interval between the two sessions. This suggests that under natural developmental conditions and within a relatively short time frame, substantial improvements in the performance of complex coordination tasks are unlikely to occur. Previous studies have reported that the motor performance in high school athletes tends to remain stable unless deliberate interventions are implemented, and the effects of natural maturation alone are generally limited [47].

In contrast, the analysis using Cramer’s V revealed statistically significant moderate-to-strong associations between initial and follow-up performances in Tasks 1, 3, and 4. These results imply that individual motor execution tendencies, whether successful or unsuccessful, are likely to be reproduced across different testing occasions. In other words, even without specific training, the motor coordination performance demonstrated intra-individual stability over time. Therefore, the “Four-Series Success Rate” may serve not only as an index for evaluating current performance levels but also as a sensitive measure for assessing personal consistency and developmental stability in upper–lower limb coordination.

By comparison, the “At Least One Series Success” criterion, which applies a more lenient threshold by considering even a single successful attempt as sufficient for success, showed a limited association between the two time points. This suggests a lack of evaluative precision and a reduced discriminatory power. However, this flexible criterion may hold contextual utility for specific populations in which generating a sense of success is critical for enhancing engagement or motivation. For example, among young children with underdeveloped motor skills, successful experiences derived from exploratory movements have been shown to facilitate the acquisition of new skills [48]. Similarly, in older adults undergoing rehabilitation, goal-oriented success is vital for fostering self-efficacy and functional recovery [49].

Turning our attention to the “average number of successful series”, findings suggest that this continuous index may serve as an effective supplementary measure for detecting nuanced performance changes. Statistically significant improvements were observed in five of the six tasks, specifically, Tasks 1, 2, 4, 5, and 6, and positive correlations between initial and follow-up performances were confirmed for Tasks 1–4. These outcomes indicated both the reproducibility of individual performance trends and the presence of a gradual improvement, even among those who initially demonstrated a low achievement. Notably, several participants who failed to complete the tasks during the first assessment increased the number of successful series at the second time point. This supports the notion that continuous-scale metrics, such as the “average number of successful series”, are capable of capturing progressive, fine-grained changes that would not be reflected in binary outcome measures based solely on full task completion.

The importance of using such sensitive and supplementary indicators has been discussed in the motor learning literature. It has been argued that skill acquisition often manifests as subtle and incremental improvements, which are not readily detectable through dichotomous success–failure evaluations [22]. Therefore, the introduction of higher-resolution performance metrics is essential for the accurate monitoring of learning trajectories.

In conclusion, this section evaluates the utility of three different performance indicators for assessing upper–lower limb coordination. The “Four-Series Success Rate” proved to be a robust and sensitive measure, capturing both the overall proficiency and intra-individual consistency. The “average number of successful series” complemented this by offering a flexible tool for identifying gradual improvements and minor performance shifts that might otherwise go unnoticed. Together, these two indices form a comprehensive framework for evaluating the coordination ability with both precision and adaptability. In contrast, the “At Least One Series Success” indicator demonstrated limited rigor as a formal assessment tool, though it may still hold value in developmental or rehabilitative contexts where motivational support through accessible success is a priority.

### 4.4. Limitations and Contributions of This Study

This study clarified how specific structural components—such as the movement direction, rhythm structure, and static control—affect success rates in coordinated limb movements and examined the potential utility of performance-based evaluation indicators derived from these components. However, this study has several limitations, which must be acknowledged. First, all tasks in this study included a uniform two-step crossing motion of the upper limbs. Consequently, it was impossible to isolate or evaluate the effects of this specific component independently. Because the presence or absence of a crossing motion was not treated as a separate variable, establishing a causal link between structural complexity and performance outcomes is inherently limited. Second, the task design was based on researchers’ hypotheses and was not quantitatively optimized using objective difficulty indices. For instance, variables such as the movement direction and timing of foot width changes were qualitatively determined. Future studies should aim to redesign the task structure using data-driven difficulty metrics to more rigorously clarify the relationship between the task composition and performance. Third, the participant sample was limited to male high school baseball players, which limits the generalizability of the findings. Factors such as age, sex, and activity levels were not varied. Given that tasks likely require physical abilities such as flexibility and jumping power, it is difficult to completely eliminate the influence of individual physical characteristics on performance. While the sample was intentionally limited to baseball players to ensure a uniformity in the training background and motor experience, this choice inherently restricted the diversity of coordination profiles, as baseball, being a unilateral-dominant sport, rarely emphasizes bilateral or rhythm-based movement training. To improve generalizability, future studies should consider including athletes from disciplines such as rowing or swimming, which involve symmetric and rhythm-focused coordination patterns and may yield different inter-limb coordination characteristics. Finally, the success or failure of each movement was determined using an observational video assessment. No physiological indices, such as electromyography or motion capture, were used. Therefore, we did not directly assess the neural mechanisms underlying coordination control. The absence of an objective physiological validation limits our understanding of these underlying processes.

In addition, owing to the extremely low number of successful performers in Tasks 5 and 6, the statistical analyses and generalization of the findings in these tasks were restricted. To enhance the reliability and validity of the task battery, future research should involve larger and more diverse participant groups across different age ranges and physical characteristics and refine the difficulty levels of each task to ensure an appropriate progression. Despite these limitations, this study makes several contributions to the literature. First, it systematically analyzes how differences in lower limb configurations affect the coordination performance when the upper limb crossing, an inherently complex spatial component, is held constant across all tasks. In particular, this study clarified the specific impact of structural elements, such as movement direction congruence and the rhythm structure, on performance outcomes, providing valuable insights into task difficulty designs for coordinated movement assessments. Second, the use of one-year follow-up data enabled a longitudinal analysis, revealing that while performance improvements were observed in some tasks, high-difficulty tasks showed limited changes. This suggests that coordination performance characteristics can be reliably and reproducibly evaluated, particularly when the performance plateaus or stabilizes in response to the task complexity.

Moreover, this task system demonstrates potential not only as an evaluation tool but also as a training modality that is adaptable to the characteristics and goals of various individuals. Flexibility in the task composition allows for both the assessment and enhancement of motor abilities. Importantly, this study introduced multiple evaluation indices that capture different aspects of coordination, suggesting their potential utility as a multifaceted foundation for future clinical, educational, and developmental research on motor coordination.

## 5. Conclusions

This study examined how structural elements such as the movement direction, rhythmic synchrony, and static postural control relate to coordination stability in upper–lower limb tasks. The findings suggest that coordination stability tends to improve when limb movement directions are congruent, the rhythmic timing is synchronized, and static control is required. In contrast, structural components such as directional incongruence, asynchronous timing, and frequent shifts in the lower limb stance appear to be associated with reduced success rates. Performance-based indicators—particularly the Four-Series Success Rate and the average number of successful series—demonstrated potential utility in capturing both the within-individual consistency and subtle longitudinal change, thereby offering a promising approach for coordination assessment. In addition, although the “At Least One Series Success” criterion, as a relatively lenient evaluative standard, showed limited precision in distinguishing performance levels, it may serve a motivational role during early-stage assessments, making it suitable for supportive evaluation contexts.

Building upon the present findings derived from frontal-plane coordination tasks, future task development may benefit from expanding into sagittal, horizontal, and rotational movement components. These additions would enable a more comprehensive investigation into the structural characteristics that influence coordination across multiple planes of motion. Such efforts would also enhance the conceptual generalizability of the current structural–performance framework, paving the way for broader applications in task design and theoretical modeling. Together, the present results provide a foundation for the continued refinement of coordination assessment systems that are both structurally informative and developmentally responsive.

## Data Availability

The data are available from the corresponding author upon request.
